# Cost analysis of total neoadjuvant therapy with 5 × 5 Gy radiation therapy versus conventional chemoradiotherapy for locally advanced rectal cancer among Peruvians

**DOI:** 10.3332/ecancer.2022.1406

**Published:** 2022-06-07

**Authors:** José Fernando Robles Díaz, Henry Olivera Changra

**Affiliations:** 1 Regional Institute of Neoplastic Diseases of the Center, Concepción, Junín 12126, Peru; 2 Los Andes Peruvian University, Huancayo, Junín 12002, Peru; 3 Continental University, Huancayo, Junín 12000, Peru

**Keywords:** costs and cost analysis, radiation dose hypofractionation, neoadjuvant therapy, rectal neoplasms, chemoradiotherapy, short-course radiation therapy

## Abstract

**Background and Objectives:**

Conventional long-course radiotherapy (LCRT) and a new paradigm of short-course radiotherapy with total neoadjuvant therapy (SCRT-TNT) are used in locally advanced rectal cancer (RC). There are few economic assessment reports available on TNT that focus on cost analysis in a country with limited funding for healthcare systems. The objective of this study was to perform a cost analysis comparing SCRT-TNT versus LCRT.

**Materials and Methods:**

In 2020–2021, a prospective registry was created to document RC patients who received neoadjuvant therapy and the costs of cancer treatments, transportation and the time patients and family members spent in the hospital. This registry outlined the direct and indirect costs of LCRT versus SCRT-TNT.

**Results:**

LCRT and SCRT-TNT regimens have direct costs that range from S/.5,993.30 to S/.27,928.36 and from S/.3,409.81 to S/.18,159.42, respectively. FOLFOX regimens are the most expensive. Administering radiotherapy in 28 3D sessions and 5 sessions of intensity-modulated radiation therapy (IMRT) or volumetric-modulated arc therapy (VMAT) sessions costs S/.2,603.88, S/.1,277.19 and S/.1,027.77, respectively. The indirect cost of FOLFOX regimens is twice that of the similar modality that combines irradiation and Oxaliplatin IV and Capecitabine VO (CAPOX). SCRT-TNT regimens with CAPOX reduce costs by at least 50%, while SCRT-TNT regimens with FOLFOX reduce costs by 32%.

**Conclusion:**

Despite using IMRT/VMAT, SCRT-TNT is a less expensive approach for patients with RC when compared to LCRT. The costs to patients using SCRT-TNT are much lower, but it is also a better option because it saves hospital resources.

## Introduction

According to GLOBOCAN 2020, colorectal cancer is the third most common type of cancer globally and the fourth most common in Peru, with incidence rates of 10% and 6.6%, respectively. Incidence-based mortality rates of colorectal cancer in Latin America and Peru are 16.6–8.2 and 11.4–5.6 per 100,000 people, respectively [1]. These rates make colorectal cancer one of the costliest cancers for healthcare systems.

It has been proven in the treatment of locally advanced rectal cancer (RC) that radiation therapy (RT) before total mesorectal excision results in low rates of pelvic recurrence [2–6]. Two radiation treatment paradigms have emerged with comparable results in overall survival and recurrence-free periods. The first paradigm is long-course chemoradiotherapy (LC-CRT), which consists of 50.4 Gy in 28 fractions with concomitant capecitabine-based chemotherapy, followed by delayed total mesorectal excision. The second paradigm is short-course radiotherapy (SCRT), which consists of 25 Gy in five sessions, followed by immediate or delayed surgical resection [7].

SCRT advocates its convenience for patients, its low cost and less acute radiation toxicities. The Trans-Tasman Radiation Oncology Group [4] and Bujko *et al*[6] have not reported significant differences in delayed toxicity between LC-CRT and SCRT. SCRT with and without pre-surgical delay were compared (surgery occurring the next week versus after a 4–8-week delay) and showed similar oncological results. But delaying surgery resulted in fewer surgical and post-operative complications [7]. This delay opens a window to provide an effective systemic therapy early in treatment that prevents the development of distant metastasis. This finding is very important as randomised trials consistently show an incidence of distant metastasis in around 30% of the patients treated for RC [3]. This new paradigm signifies progress towards a useful systemic therapy with oxaliplatin-based CTx to prevent the development of distant metastasis. Being able to administer all adjuvant therapies preoperatively would, therefore, be ideal. This concept is known as total neoadjuvant therapy (TNT). The main objective of TNT is to prevent distant metastasis by instructing patients to use an effective systemic therapy in the first stages of the disease. Other TNT benefits are a better local response, which may appear as both clinical and complete pathological responses, better tolerance of and compliance with treatment, reduced overall time required to complete treatment and earlier stoma closure [8, 9].

There have been few economic assessment reports on TNT to date. Examining the optimal neoadjuvant regimens for patients with RC is thus important, as is considering cost analysis in a country where healthcare system funding is limited. The objective of this study was, therefore, to perform a cost analysis to compare SCRT-TNT versus LCRT in patients with locally advanced rectal cancer.

## Materials and methods

In 2020–2021, the clinical features of RC patients who received neoadjuvant therapy were prospectively recorded using medical histories. Transportation expenses were collected using patient questionnaires on their daily expenses for travelling to the institute. Medical costs were obtained from medical histories and pharmacy records. The medications and medical devices used to prepare and administer chemotherapy and radiation treatments were also identified during this process and the involved healthcare professionals were interviewed to determine the duration. This study was approved by the Institutional Ethics Committee in advance and only analysed patients who provided complete data.

### Estimated cost

A spread sheet template was created using the averages of patients who received neoadjuvant therapy, to outline the cost of LCRT and SCRT-TNT treatments. The template included the direct and indirect medical costs associated with the time healthcare professionals worked, equipment and infrastructure, and the medications and supplies used in Peruvian currency (Peruvian Nuevo Sol, S/.). The template also included non-medical costs associated with the time patients and relatives spent in the facility, including travel time from their homes.

Data analysis was carried out for patients treated at the institute with tumour staging (≥3) NxM0, who completed treatment and were granted full access to costs. If radiation was part of the LC-CRT, the three-dimensional (3D) technique was used, but if radiation was part of SCRT, inverse planning was used (intensity-modulated radiation therapy (IMRT)/volumetric-modulated arc therapy (VMAT)).

### Regimens

This study compared these treatment regimens (Table 1): 1) CTx-RT in 28 sessions with 3D techniques, oral capecitabine and 8 courses of adjuvant Oxaliplatin IV and Capecitabine VO (CAPOX) therapy (LCRT 3D + CAPOX); 2) CTx-RT in 28 sessions with 3D techniques, oral capecitabine and 12 courses of adjuvant FOLFOX therapy (LCRT 3D + FOLFOX); 3) RT with IMRT in 5 sessions, followed by 6 courses of CAPOX (SCRT IMRT + CAPOX); 4) RT with VMAT in 5 sessions, followed by 6 courses of CAPOX (SCRT VMAT + CAPOX); 5) RT with IMRT in 5 sessions, followed by 9 courses of FOLFOX (SCRT IMRT + FOLFOX); 6) RT with VMAT in 5 sessions VMAT, followed by 9 courses of FOLFOX (SCRT VMAT + FOLFOX). Notably, FOLFOX involves 4 days in hospital per course, while CAPOX involves 6 hours of chemotherapy in a day hospital.

### Direct cost

Direct costs were calculated using medication costs shared by the institutional pharmacy cost centre and the costs of equipment and infrastructure use and procedures for consultations, simulations, target volume delineation and treatment planning. Costs related to healthcare staffing were determined using the healthcare professionals’ monthly payroll, calculating per-minute values. The cost of mesorectal excision was not analysed as it did not vary between LCRT versus SCRT-TNT nor were toxic effects analysed as there were no significant adverse effects in most cases.

### Indirect cost

Indirect costs to patients included those associated with their hospital stays, calculated from their admission to their discharge. Costs related to transportation were determined using average patient costs for travelling from their homes to the hospital. Our analysis included costs associated with companions who accompanied patients because the therapies study participants received normally affect patients independence. A population-based, per-hour-worked reference value for the central macro-region was used to calculate patient or household loss of productivity due to being in hospital to either receiving cancer treatments or as companions, respectively, and therefore missing work [10].

### Statistical analysis

All data and statistical analyses were carried out using SPSS version 21. Descriptive statistics are presented as means or proportions.

## Results

After reviewing the 2020–2021 case histories, 38 were found for patients with neoadjuvant regimens that contained complete information for the study. Table 2 shows patient characteristics and Table 3 outlines the unit cost of the resources used for comparative projections.

The LCRT 3D + CAPOX, LCRT 3D + FOLFOX, SCRT IMRT + CAPOX, SCRT VMT + CAPOX, SCRT IMRT + FOLFOX and SCRT VAMT + FOLFOX regimens have direct costs of S/.5,993.30, S/.27,928.36, S/.3,659.72, S/.3,409.81, S/.18,159.42 and S/.17,909.50, respectively. FOLFOX regimens are the most expensive. Administering radiotherapy in 28 3D sessions and 5 IMRT or VMAT sessions costs S/.2,603.88, S/.1,277.19 and S/.1,027.77, respectively. Planning treatment thus has an increased value over 3D of S/.324.54 for VMAT and S/.341.53 for IMRT. Likewise, the cost of all 3D teletherapy sessions (28) is triple the cost of 5 IMRT sessions and 5 times that of 5 VMAT sessions (Table 4).

The indirect cost of regimens that include FOLFOX is higher, amounting to twice that of regimens that use the same radiation technique but include CAPOX. SCRT-TNT regimens with CAPOX reduce costs by at least 50%, while SCRT-TNT regimens with FOLFOX reduce costs by 32%. The cost of lost household productivity for LCRT 3D + CAPOX, LCRT 3D + FOLFOX, SCRT IMRT + CAPOX, SCRT VMT + CAPOX, SCRT IMRT + FOLFOX and SCRT VAMT + FOLFOX is S/.914.48, S/.1,721.3, S/.428.25, S/.428.25, S/.1,091.87 and S/.1,091.87, respectively (Table 4).

**Figure 1.**
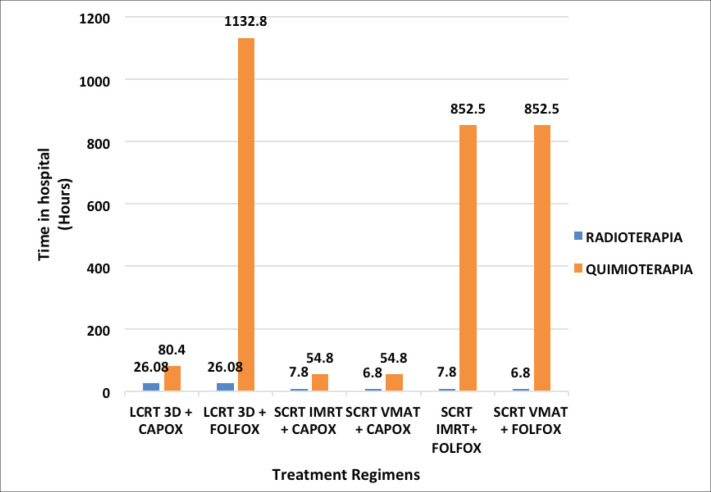
Time that patients spend in hospital for each regimen.

Patients spend over 850 hours in the hospital for FOLFOX regimens versus less than 100 hours for CAPOX regimens. Moreover, SCRT VMAT + CAPOX is the shortest of the SCRT-TNT regimens, with 61.6 hours in the hospital (Figure 1).

**Figure 2.**
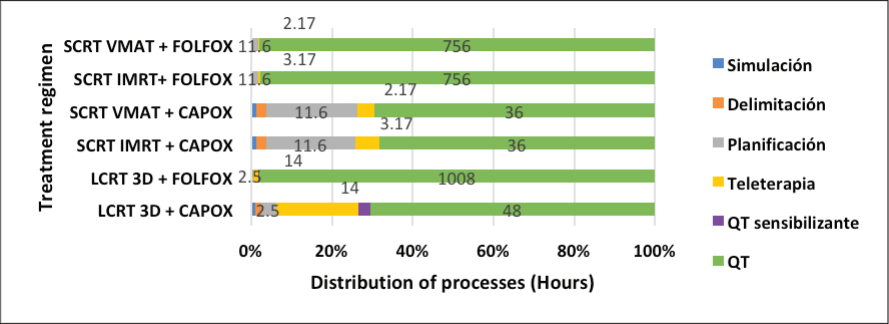
Comparison of time for each process by regimen. Sensitising QTx: Capecitabine VO-based chemotherapy, used during QTx-RT. QTx: Neoadjuvant or adjuvant chemotherapy regimen in the form of CAPOX or FOLFOX.

The distribution of these processes shows that SCRT-TNT regimens only take around 3 hours to administer the total prescribed radiation, while LCRT regimens take a minimum of 14 hours. Similarly, full-dose FOLFOX chemotherapy in the SCRT-TNT regimen takes 756 hours versus 1,008 hours in the LCRT regimen. CAPOX chemotherapy takes 36 hours in the SCRT-TNT regimen versus 48 hours in the LCRT regimens (Figure 2).

## Discussion

The institute is a public facility located 284 km from the national capital and covers most of the central Peruvian Andes. Patients with rectal cancer have comprehensive health insurance, which subsidises the direct costs of medical care. For neoadjuvant therapy, the Peruvian government pays the cost of medication, associated supplies, equipment and infrastructure and the fee of healthcare professionals. However, funding for neoadjuvant therapy is limited and requires making decisions. These choices must reflect the efficacy of treatments and the direct and indirect costs associated with the patient’s recovery to make more cancer treatments available [11, 12].

We have known, since the Gastrointestinal Tumour Study Group research was published in the *New England Journal of Medicine* in 1985, that surgery is insufficient for locally advanced RC. This study showed a clear benefit to adding RT and concomitant CTx around 5 weeks after surgery (adjuvant) [13]. A 1997 study in Sweden compared a preoperative (neoadjuvant) 25 Gy dose of RT administered in 5 sessions (SCRT), followed by surgery within 1 week versus surgery alone. The study found that SCRT led to better local control and greater overall survival [14].

It seems counterintuitive that SCRT with 25 Gy is equivalent to CTx-RT with twice the dose. But a radiobiological analysis shows that these doses are equivalent as the 25 Gy is administered in doses of 5 Gy per session versus the conventional 1.8–2.0 Gy per session. Furthermore, completing SCRT treatment requires only 1 week versus 5–5.5 weeks.

Analysis that uses a linear quadratic model and accounts for total treatment time shows that SCRT delivers the equivalent of 35.7 Gy versus 34.4 Gy in LCRT treatment [9, 15].

To date, we have understood that both modalities (SCRT and LCRT) provide comparable local results. Before the TNT concept emerged, treatments combined CTx, which used induction and/or consolidation cycles, with standard CTx-RT before surgery to increase pathological degradation and act on occult micrometastatic diseases [16]. The PRODIGE23 study confirmed this approach, by finding a 3-year metastasis-free survival rate of 78% [17]. Finally, the RAPIDO study combined SCRT with high-dose CTx before mesorectal excision. RAPIDO randomised patients with high-risk characteristics to TNT with oxaliplatin for 18 weeks versus CTx-RT adjuvant oxaliplatin treatment for 24 weeks. The treatment was well tolerated in the TNT arm, with these adverse events of grade 3 or higher: diarrhoea in 17.6% of the group; vascular disorders in 8.5%; and all other adverse events affecting lesser than 5%. The primary focus was treatment-related failure, which was 23.7% in the group receiving TNT versus 30.4% in the control group (*p* = 0.019). The complete pathological response was duplicated in the TNT group, reaching 28.4% versus 14.3% (*p* < 0.001). Distant metastasis occurred in 20% of the patients in the TNT group versus 26.8% of the patients in the CTx-RT group (*p* = 0.005) [8]. The institute, given proof of better clinical results and a better toxicity profile [8, 9, 16–18], has since 2021 opted to follow RAPIDO study guidelines and manage patients using TNT treatment regimens, such as SCRT-TNT, and to abandon the LCRT regimen.

Many published studies have examined preoperative CTx-RT regimens that have been used widely in clinical practice. However, research on economic analysis is rarely reported. This is the first Latin American study to analyse the costs of TNT (SCRT with CTx) and LCRT for patients with advanced RC.

Our data show that SCRT-TNT regimens are less expensive than LCRT. The SCRT IMRT/VMAT + CAPOX treatment plan results in the lowest direct costs. Teletherapy reduces the cost by four-fifths (from 28 to 5 sessions) and a course of CAPOX treatment is administered in about 6 hours in an outpatient setting. On the contrary, FOLFOX requires hospitalising the patient for 4 days per course, raising its cost. However, unsupervised outpatient CTx with capecitabine involves a risk of irregular treatment and can be inappropriate for some patients, leading to worse outcomes [19–21]. Administering SCRT requires specialised techniques, such as IMRT or VMAT, which costs less, and there is no marked difference between them. Raldow *et al* [22] found that SCRT was more cost-effective than LCRT. But the study also analysed the location and found that LCRT was more cost-effective in tumours in the distal third of the rectum. However, in this study, patients underwent surgery after receiving RT. It is likely that the interval after radiotherapy, which we provide our patients before surgery under the RAPIDO oxaliplatin protocol, would be more favourable even in distal tumours, comparable to cost-effectiveness in other sites [8]. Wang *et al* [23] compared SCRT followed by CTx (TNT) to LCRT, and found that the general costs of SCRT-TNT and LC-CRT were $78,937 and $38,140, with an efficacy of 29.92 and 22.99 quality-adjusted life months (QALM), respectively. SCRT is thus a rather cost-effective strategy. Moreover, the study found that CTx costs were always higher in both SCRT-TNT and LCRT ($7,064.01 and $5,670.94, respectively), while RT costs were $3,693.70 and $5,589.70, respectively. These findings are consistent with our results, showing that the costs of CT are up to 2.3 or up to 16 times higher when using CAPOX or FOLFOX, respectively (Table 4).

Our study shows that indirect costs are lower for all TNT modalities. The difference in the loss of productivity and the use of transportation is notable (Table 4). This is because patients need fewer hospital visits, and thus spend fewer total hours in teletherapy or chemotherapy (Figure 1). Results from comparing CAPOX and FOLFOX are consistent with those of Lin *et al* [24], which found that FOLFOX generates higher indirect costs due to the loss of productivity for the patient and the relatives who accompany them. An evaluation of only these two types of indirect costs found that they represent 36% and 8% of the annual household income (S/.13,628) [10] with LC-CRT 3D + FOLFOX and SCRT IMRT/VMAT + CAPOX regimens, respectively (Table 4). This result is worrisome, due to the risk of incurring catastrophic costs and increasing barriers to treatment compliance [25, 26].

Hanly *et al* [27] showed that radiation therapy cost between €2,080 (5-fraction cycle) and €3,609 (25-fraction cycle) per patient. Costs were higher during the treatment planning phase for a short course (€1,217; 58% of the total cost), but were still higher in the long-cycle teletherapy phase (€1,974; 60% of the total cost). These results are consistent with our distribution analysis for hours involved professionals worked. They also show how complex the planning process is, but with fewer hours of teletherapy in the SCRT regimen (Figure 2). The study accounted for simultaneous variations in treatment time, capacity utilisation rates and the number of linear accelerator personnel, and found that the baseline cost fell by 20% for 5 fractions (€1,660) and 35% for 25 fractions (€2,354). In our analysis, the minimum required number of personnel for the Elekta® Synergy full linear accelerator (LINAC) was used: two medical technologists. Staffing costs could thus not have been reduced further. Perhaps using another LINAC that optimises treatment speed through gantry mobility and MLC motion, such as the Varian® Halcyon [28, 29], could significantly lower teletherapy costs.

Our analysis has limitations. First, we made some simplifying assumptions regarding the natural history and treatment of the disease. Second, it is possible we did not account for differences in delayed toxic effects as follow-up data are limited. However, the absolute rate of these effects seems lower for SCRT-TNT according to clinical trials [7, 8, 17]. Third, adverse events were underreported.

Our study is one of the few to evaluate the costs of LCRT and SCRT-TNT treatments that have incorporated patient weight, direct costs and the indirect cost of time to patients and their households. Moreover, this is the first report that includes clinical and economic oncologic data from a Peruvian public hospital.

## Conclusion

In patients with locally advanced RC, SCRT-TNT is a less expensive approach than LCRT, despite using IMRT/VMAT. Using SCRT-TNT is also preferable because hospitals save resources and patients incur much lower indirect costs. Among the SCRT-TNT modalities, the use of CAPOX is much less expensive because it is an outpatient treatment. However, its usefulness has to be assessed to reflect the patient’s profile.

## Funding

No funding was received.

## Conflicts of interest

The authors declare that they have no competing interests.

## Figures and Tables

**Table 1.** Description of treatment regimens.

Regimens		Pre-surgical	Post-surgical
LONG	LCRT 3D + CAPOX	QTx-RT: 56 Gy in 28 sessions in 3D concurrent with oral capecitabine	8 courses of CAPOX
	LCRT 3D + FOLFOX	QTx-RT: 56 Gy in 28 sessions in 3D concurrent with oral capecitabine	12 courses of FOLFOX
SHORT-TNT	SCRT IMRT + CAPOX	RT -> QTx: 25 Gy in 5 sessions in IMRT, followed by 6 courses of CAPOX	-
	SCRT VMAT + CAPOX	RT -> QTx: 25 Gy in 5 sessions in VMAT, followed by 6 courses of CAPOX	-
	SCRT IMRT+ FOLFOX	RT -> QTx: 25 Gy in 5 sessions in IMRT, followed by 9 courses of FOLFOX	-
	SCRT VMAT + FOLFOX	RT -> QTx: 25 Gy in 5 sessions in VMAT, followed by 9 courses of FOLFOX	-

RT: Radiotherapy. QTx: Chemotherapy. CAPOX (Oxaliplatin IV and Capecitabine VO). FOLFOX (Oxaliplatin IV, Fluorouracil IV and Leucovorin IV). IMRT: Intensity-modulated radiotherapy. VMAT: Volumetric intensity-modulated arc therapy

**Table 2.** Characteristics of patients and companions.

Category	*N*	%	Mean	Standard deviation
Patient				
Age	38		52.0	4.1
Gender				
Male	28	73.7		
Female	10	26.3		
Body area	38		1.5	0.0
Companion				
Yes	38	100.0		
No	0	0.0		
Age	38		24.6	9.5
Working	10	26.3		
Unemployed	28	73.7		
Monthly household Income (Peruvian Nuevo Sol S/.)	38		1031.7	100.9

**Table 3.** Unit costs associated with radiotherapy and chemotherapy.

Phase	Resources	Soles^b^
Radiotherapy	Radiation oncologist^a^	0.871
	Medical technologist^a^	0.527
	Nurse^a^	0.577
	Nursing technician^a^	0.577
	Medical physicist^a^	0.556
	Tomography simulator^a^	0.320
	Laser alignment system^a^	0.070
	Simulation infrastructure^a^	0.019
	Simulation equipment^a^	0.043
	Immobilisation accessory^a^	0.000
	Software^a^	0.230
	3D planning infrastructure and equipment^a^	0.003
	IMRT/VMAT planning infrastructure and equipment^a^	0.011
	3D teletherapy equipment^a^	2.270
	3D teletherapy infrastructure^a^	0.016
	IMRT/VMAT teletherapy equipment^a^	2.300
	IMRT/VMAT teletherapy infrastructure^a^	0.016
Chemotherapy	Medical oncologist^a^	0.871
	Nurse^a^	0.577
	Nursing technician^a^	0.311
	Capecitabine tablet 500 mg tablets per unit	0.750
	Oxaliplatin injection 100 mg per unit	29.000
	Fluorouracil injection 500 mg per unit	11.160
	Omeprazole 20 mg cap per unit	0.080
	Omeprazole injection 40 mg per unit	0.850
	Metoclopramide tablet 10 mg per unit	0.060
	Metoclopramide injection 5 mg/ml per unit	0.320
	Ondansetron injection 2 mg/ml per unit	2.600
	Ondansetron tablet 8 mg tablets per unit	1.110
	Calcium folinate injection 50 mg	16.740
	Infusion set	6.850
	Heparin syringe	2.500
	20 ml syringe	0.390
	Sterile gloves	2.220
	Protective suit for 6 hours	6.880
	Saline solution 250 mL	3.380
	Gauze	1.800
	Cotton swabs	0.300
	Surgical tape	0.230
	Alcohol	0.010
	Hospitalisation infrastructure^a^	0.032
	Chemotherapy infrastructure^a^	0.010

a Cost equivalent to 1 minute of utility

b 1.0 is equal to 1 sol

**Table 4.** Comparison of direct and indirect costs by treatment regimens.

Direct cost								
Regimens			LCRT		SCRT-TNT			
Phase		Category	LCRT 3D + CAPOX	LCRT 3D + FOLFOX	SCRT IMRT + CAPOX	SCRT VMAT + CAPOX	SCRT IMRT+ FOLFOX	SCRT VMAT + FOLFOX
NEOA	RT	Consultation	75.90	75.90	37.95	37.95	37.95	37.95
		Simulation	107.96	107.96	107.96	107.96	107.96	107.96
		Delineation	58.27	58.27	77.69	77.69	77.69	77.69
		Planning	89.75	89.75	431.28	414.29	431.28	414.29
		Teletherapy	2,271.99	2,271.99	622.30	389.38	622.30	389.38
		Subtotal	2,603.88	2,603.88	1,277.19	1,027.27	1,277.19	1,027.27
	CT	Consultation	79.39	79.39	158.78	158.78	218.32	218.32
		CT Sensitizer	146.54	146.54	0.00	0.00	0.00	0.00
		CAPOX	0.00	0.00	2,223.76	2,223.76	0.00	0.00
		FOLFOX	0.00	0.00	0.00	0.00	16,663.91	16,663.91
		Subtotal	225.93	225.93	2,382.54	2,382.54	16,882.23	16,882.23
A	CT	Consultation	198.47	238.17	0.00	0.00	0.00	0.00
		Chemotherapy	2,965.01	2,091.55	0.00	0.00	0.00	0.00
		Subtotal	3,163.49	25,098.55	0.00	0.00	0.00	0.00
Total per patient (Peruvian Nuevo Sol S/.)			5,993.30	27,928.36	3,659.72	3,409.81	18,159.42	17,909.50
Total per patient (dollars)*			1,498.32	6,982.09	914.93	852.45	4,539.86	4,477.38
**INDIRECT COST**								
**REGIMENS**			**LCRT**		**SCRT - TNT**			
**Phase**	**Category**		**LCRT 3D + CAPOX**	**LCRT 3D + FOLFOX**	**SCRT IMRT + CAPOX**	**SCRT VMAT + CAPOX**	**SCRT IMRT+ FOLFOX**	**SCRT VMAT + FOLFOX**
NEOA	Loss of productivity due to consultations and treatment		199.22	154.27	370.29	364.37	1,873.31	1,867.40
	Transportation		445.50	445.50	297.00	297.00	378.00	378.00
	Loss of household productivity		483.86	429.44	428.25	428.25	1,091.87	1091,87
	Subtotal		1,013.08	913.70	1,018.54	1,012.62	3,245.18	3,239.27
A	Loss of productivity due to consultations and treatment		430.62	2,413/36	0.00	0.00	0.00	0.00
	Transportation		243.00	216.00	0.00	0.00	0.00	0.00
	Loss of household productivity		430.62	1,291.86	0.00	0.00	0.00	0.00
	Subtotal		1,104.24	3,921.22	0.00	0.00	0.00	0.00
Total per patient (Peruvian Nuevo Sol S/.)			2,232.82	4,950.42	1,095.54	1,089.62	3,343.18	3,337.27
Total per patient (dollars)*			558.20	1,237.61	273.88	272.41	835.80	834.32

LCRT: Radiation regimen in 28 sessions and after surgery, adjuvant with 8 courses of CAPOX or 12 courses of FOLFOX. SCRT-TNT: Radiation regimen in 5 sessions, followed by 6 courses of CAPOX or 9 courses of FOLFOX. NEOA: Neoadjuvant. ADY: Adjuvant. RT: Radiotherapy. QTx: Chemotherapy. LCRT 3D + CAPOX: QTx-RT, followed by 8 courses of CAPOX (oxaliplatin IV and capecitabine VO). LCRT 3D + FOLFOX: QTx-RT, followed by FOLFOX (oxaliplatin IV, fluorouracil IV and leucovorin IV). SCRT IMRT + CAPOX: 25 Gy/5Fx in IMRT, followed by CAPOX (oxaliplatin IV and capecitabine VO). SCRT VMAT + CAPOX: 25 Gy/5Fx in VMAT, followed by CAPOX (oxaliplatin IV and capecitabine VO. SCRT IMRT + FOLFOX: 25 Gy/5Fx in IMRT, followed by FOLFOX (oxaliplatin IV, fluorouracil IV and leucovorin IV). SCRT VMAT + FOLFOX: 25 Gy/5Fx in VMAT, followed by FOLFOX (oxaliplatin IV, fluorouracil IV and leucovorin IV). *: Equivalent in US dollars (4.0)
